# PP2A/B55 and Fcp1 Regulate Greatwall and Ensa Dephosphorylation during Mitotic Exit

**DOI:** 10.1371/journal.pgen.1004004

**Published:** 2014-01-02

**Authors:** Nadia Hégarat, Clare Vesely, P. K. Vinod, Cory Ocasio, Nisha Peter, Julian Gannon, Antony W. Oliver, Béla Novák, Helfrid Hochegger

**Affiliations:** 1 Genome Damage and Stability Centre, School of Life Sciences, University of Sussex, Falmer, Brighton, United Kingdom; 2 Oxford Centre for Integrative Systems Biology, Department of Biochemistry, University of Oxford, Oxford, United Kingdom; 3 Genome Stability, Cancer Research UK, Clare Hall Laboratories, South Mimms, Herts, United Kingdom; The University of North Carolina at Chapel Hill, United States of America

## Abstract

Entry into mitosis is triggered by activation of Cdk1 and inactivation of its counteracting phosphatase PP2A/B55. Greatwall kinase inactivates PP2A/B55 via its substrates Ensa and ARPP19. Both Greatwall and Ensa/ARPP19 are regulated by phosphorylation, but the dynamic regulation of Greatwall activity and the phosphatases that control Greatwall kinase and its substrates are poorly understood. To address these questions we applied a combination of mathematical modelling and experiments using phospho-specific antibodies to monitor Greatwall, Ensa/ARPP19 and Cdk substrate phosphorylation during mitotic entry and exit. We demonstrate that PP2A/B55 is required for Gwl dephosphorylation at the essential Cdk site Thr194. Ensa/ARPP19 dephosphorylation is mediated by the RNA Polymerase II carboxy terminal domain phosphatase Fcp1. Surprisingly, inhibition or depletion of neither Fcp1 nor PP2A appears to block dephosphorylation of the bulk of mitotic Cdk1 substrates during mitotic exit. Taken together our results suggest a hierarchy of phosphatases coordinating Greatwall, Ensa/ARPP19 and Cdk substrate dephosphorylation during mitotic exit.

## Author Summary

Greatwall kinase regulates a switch between kinase and phosphatase activity during mitotic entry and constitutes an essential element of mitotic control. This control system is further complicated by the fact that Greatwall itself is regulated via phosphorylation and acts by phosphorylating its substrates ENSA and ARPP19. A missing link in this central mitotic switch is the phosphatase that counteracts Greatwall and its target Ensa. We have used mathematical modeling and experimental validation to identify these phosphatases. We demonstrate genetic evidence that Greatwall phosphorylation is counteracted by PP2A/B55, while Fcp1 regulates ENSA dephosphorylation. Based on these findings we present a new model for the regulation of mammalian cell division.

## Introduction

Phosphorylation of more than thousand proteins by Cdk1 and other mitotic kinases drives entry into mitosis [1], [2]. As cells exit mitosis, these post-translational modifications have to be removed by phosphatases. Mitotic kinase and phosphatase activity appears to be inversely regulated to avoid futile cycles of phosphorylation and dephosphorylation [3], [4]. Moreover, mitotic kinases themselves are regulated by phosphorylation and dephosphorylation resulting in a complex feedback system of cell cycle control [5]. Cdk1 is negatively regulated by phosphorylation at Thr14/Tyr15 by Wee1 and Myt1 kinases and dephosphorylation of this site by the Cdc25 phosphatase constitutes the decision point to enter mitosis [6]. Cdk1 actively participates in its own activation by negatively regulating its inhibitor Wee1 [7]–[9] and positively regulating its activator Cdc25 [10]. In Xenopus egg extracts this switch is counteracted by the phosphatase PP2A/B55δ [11], [12] suggesting that inhibition of PP2A/B55δ is an intrinsic element of the G2/M transition. This is achieved by Greatwall kinase (Gwl) [13]–[15] that phosphorylates and activates the PP2A/B55δ inhibitors Endosulfin (Ensa) and ARPP19 [16]–[19]. The Gwl phosphorylation motive FDSGDY is identical in Ensa and ARPP19 and thus detectable with the same phospho-specific antibody. For simplicity we will refer in our analysis to Ensa/ARPP19, because it is impossible to distinguish between the phosphorylation of the two proteins with specific antibodies. Depletion of Gwl kinase in Xenopus mitotic extracts results in rapid Cdk1 inactivation and exit from mitosis, while Gwl depletion in interphase extracts blocks Cdk1 Thr14/Tyr15 dephosphorylation and mitotic entry [14], [15]. In human cells Gwl kinase depletion causes a delay in mitotic entry, reduces Cdk substrate phosphorylation and results in chromosome alignment defects and aberrant mitotic exit [20], [21]. Cdk1 phosphorylates Gwl at multiple sites and is required for its activation [22]. Thus, Cdk1 and Gwl activation are locked in a complex feedback loop at the G2/M transition.

To gain a more precise understanding of this switch-like transition, the phosphatases that target Gwl itself and Ensa/ARPP19 have to be identified. Inactivation of these phosphatases is likely to play a major role in the initiation of the Cdk1 activation loop. Moreover, the reactivation of these phosphatases is likely to be a crucial element during mitotic exit. The identity of the major Cdk1 counteracting phosphatase in mammalian cells is also still under debate. Current models propose that PP2A/B55δ is not only required for the Cdk1 activation loop, but also to directly dephosphorylate mitotic substrates during mitotic exit [3], [23]. Thus, PP2A/B55δ has been proposed to be the major Cdk1 counteracting phosphatase during mitotic exit in Xenopus egg extracts, equivalent to the function of Cdc14 phosphatase in budding yeast. This hypothesis is based on the observation that co-depletion of Wee1, Myt1 and Gwl causes mitotic exit from Xenopus egg extracts despite persistent high Cdk1 activity [24]. Conversely, B55δ depletion does not block Cdk1 substrate dephosphorylation, when mitotic exit is triggered by Cdk1 inhibition in Xenopus egg extracts [12] and PP1 has also been implicated to act as a major mitotic exit phosphatase [25]. In human cells PP2A/B55α has been implicated in regulating mitotic exit, but B55α depletion shows only a delay but not a block of Cdk substrate dephosphorylation following Cdk inactivation [26]. Thus, the network of phosphatases that counteract Gwl, Ensa/ARPP19 and Cdk phosphorylation during mitotic exit remains to be determined.

## Results

### 1) Modelling Gwl and Cdk1 phosphorylation dynamics at the G2/M transition and mitotic exit

**Figure 1:**
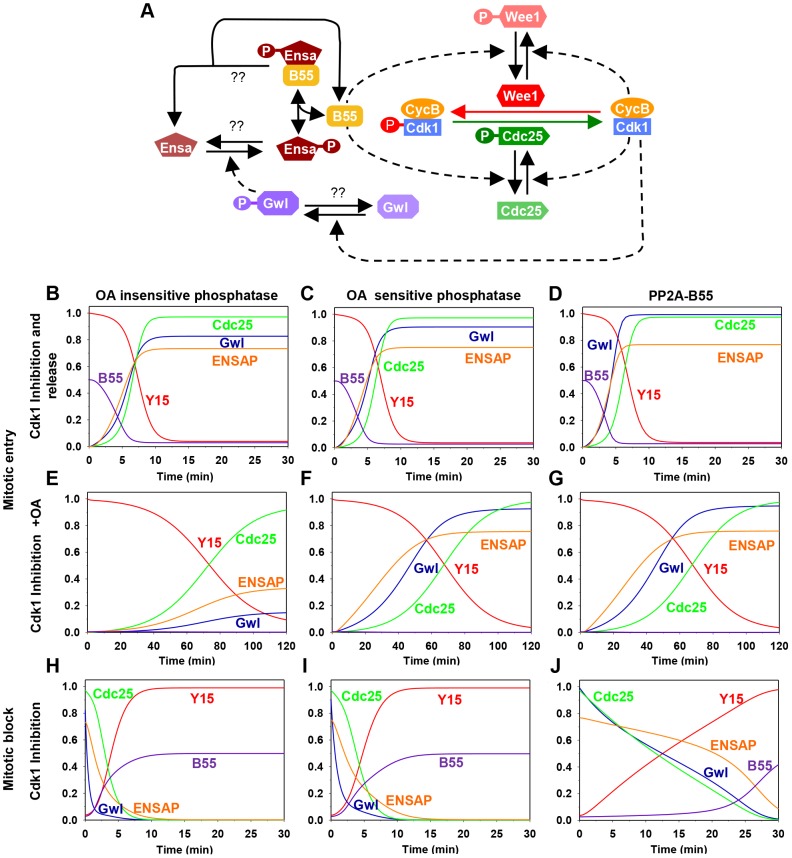
The regulatory network and the dynamics of the mitotic switch. (**A**) Model of the Cdk1 activation switch. Cdk1 activity is regulated by inhibitory Tyr15 phosphorylation modulated through the activities of Wee1 and Cdc25. The activity of these modifying enzymes are regulated both directly and indirectly (through Gwl, Ensa/ARPP19 and PP2A/B55) by Cdk1. (**B–J**) Simulation of mitotic entry and exit using a mathematical model of the regulatory network with the assumption included that Gwl is dephosphorylated by an OA-insensitive phosphatase (left panels), by an OA-sensitive phosphatase (middle panels), or specifically by PP2A/B55 (right panels). The simulation of mitotic entry is shown from the initial condition obtained using Cdk1 inhibition and either removal of Cdk1 inhibition (B–D) or PP2A inhibition by OA (E–G) promotes mitotic entry. The mitotic exit (H–J) is shown from the initial condition corresponding to metaphase state and Cdk1 inhibition promotes mitotic exit.

To gain insight into the dynamics of the Gwl/Cdk1 feedback loop during mitotic entry and exit we performed mathematical modelling (see Material and Methods) of the Cdk1 regulatory network shown on Fig. 1A. This network has multiple positive circuits (positive and double-negative feedback loops) including the antagonism between Cdk1 and PP2A/B55 that plays a key role in the switch-like transitions at G2/M and mitotic exit. PP2A/B55 inhibits Cdk1 through the Tyr-modifying enzymes while Cdk1 down-regulates PP2A/B55 activity through Gwl and Ensa/ARPP19 activation. As a consequence of these feedback loops the network has two qualitatively different states corresponding to G2 and M phases. In G2, both Gwl and Cdk1 are inactive while PP2A/B55 is active, but in M phase the opposite is true. Based on this information, we built mathematical models to analyse the impact of different Gwl phosphatases on the dynamics of mitotic entry and exit (Fig. S1). Inactivation of Cdk1 (by chemical inhibition) stabilizes the G2 phase (high Cdk1 Tyr15 phosphorylation, inactive Gwl and active PP2A/B55), allowing Cyclin B to accumulate above the threshold normally required for G2/M transition. Mitotic entry can be triggered by either terminating Cdk1 kinase inhibition, or by inactivation of PP2A by Okadaic acid (OA). In this scenario Cdk1 re-activation destabilizes the G2 state and initiates rapid transition into M phase characterized by Tyr15 dephosphorylation, Gwl activation and PP2A/B55 inhibition (Fig. 1B–D). Conversely, PP2A inhibition causes only a slow decrease in Cdk1 Tyr15 phosphorylation because phosphorylation of Tyr-modifying enzymes is compromised by low Cdk1 activity (Fig. 1E–G). If the Gwl phosphatase is insensitive to OA, PP2A inhibition should not induce phosphorylation of these proteins, because Cdk1 activity remains low and the counteracting phosphatases are active (Fig. 1E). In contrast, if Gwl is targeted by an OA sensitive phosphatase, Cdk1 Tyr-dephosphorylation should be accompanied by Gwl phosphorylation (Fig. 1F and G). For simplicity we presume constitutive activity of the Ensa/ARPP19 phosphatase. Activation of Gwl overcomes this activity and results in Ser67 phosphorylation. Furthermore, the model predicts that Gwl phosphorylation precedes Tyr15-dephosphorylation (Fig. 1F and G) because the latter requires prior phosphorylation of Cdc25 and Wee1 to remove the inhibitory Tyr15 phosphorylation. To obtain further information about Gwl phosphatases we also simulated mitotic exit triggered by Cdk1 inactivation (Fig. 1). The model suggests that Gwl will be instantaneously dephosphorylated after Cdk1 inactivation, if a constitutive phosphatase is responsible for its inactivation (Fig. 1H and I). If the Gwl phosphatase is directly participating in a double negative feedback loop (such as the M-phase inactive phosphatase PP2A/B55) then Gwl inactivation is a slow process to allow for time of phosphatase reactivation (Fig. 1J). Thus, our model of the kinetics of mitotic entry and exit potentially revealed important information about the nature of the Gwl phosphatase, which can be tested experimentally.

### 2) Detecting an essential Cdk site in the putative T-loop of Gwl kinase

**Figure 2:**
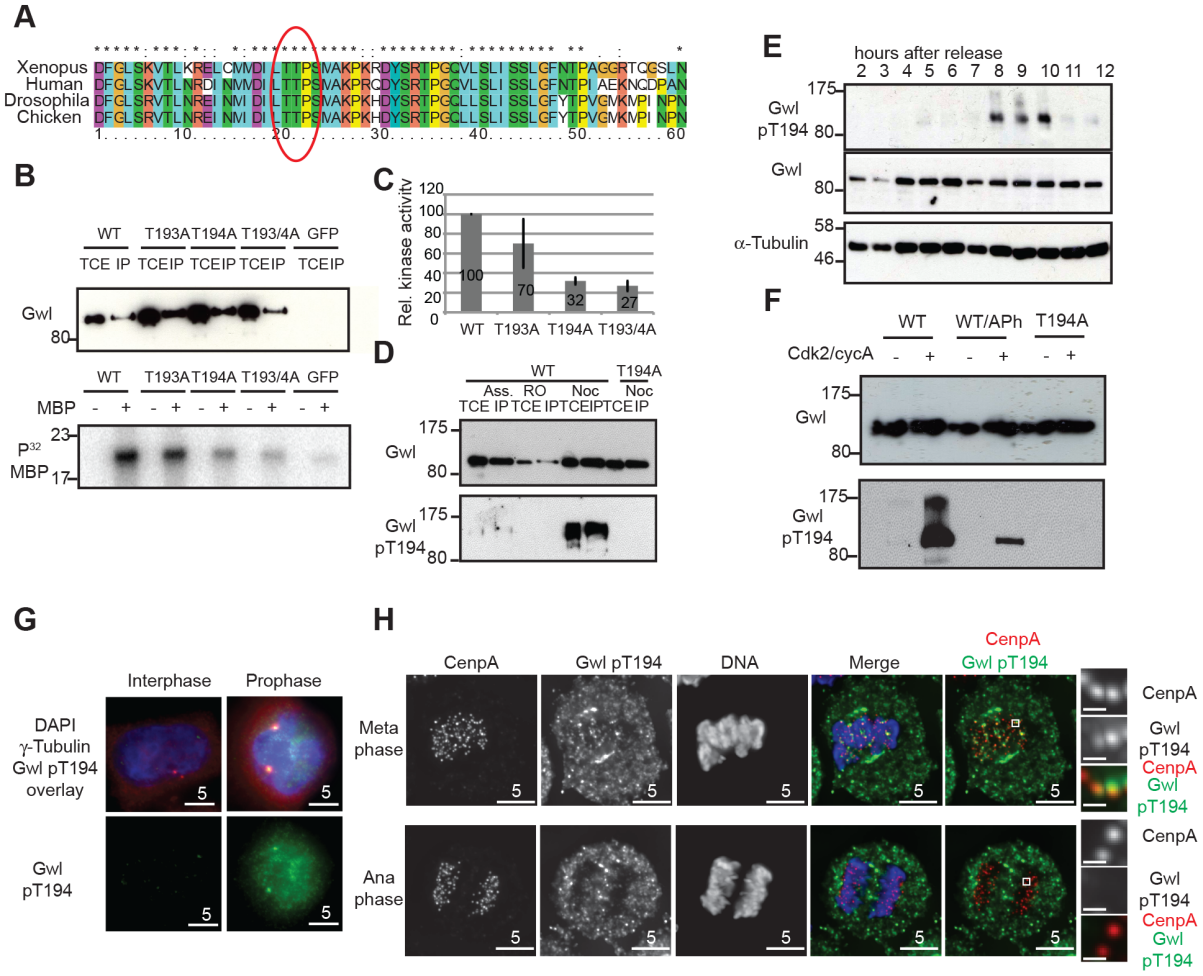
Gwl Thr194 is an essential Cdk1 phosphorylation site. (**A**) Multiple amino-acid sequence alignment of Gwl sequence following the DFG motif. (**B**) IP/kinase assay of transiently expressed Flag-tagged WT and T193A/Thr194A mutant Gwl from nocodazole arrested HEK 293T cells using MBP as a substrate. (TCE total cell extract, IP immuno-precipitate) (**C**) Quantification of kinase assays as shown in (B). The average of 3 independent experiments was calculated and the error bars show the standard deviation between the different assays. (**D**) IP/Western of transiently expressed Flag WT and Thr194A mutant Gwl from asynchronous, RO3306, or nocodazole arrested HEK 293T cells. (**E**) Immunoblot of *HeLa* cell extracts following a double thymidine release sampled at indicated time points. Cell cycle progression was simultaneously monitored by PI staining and FACS analysis (see Fig. S2B). The cells passed through mitosis between 8 and 10 hours following release as indicated. (**F**) Gwl phosphorylation by CycA/Cdk2 *in vitro*. Flag WT and Thr194A Gwl was transiently expressed and purified from asynchronous HEK 293T cells and incubated with recombinant CycA/Cdk2, following treatment with alkaline phosphatase (aPh) in the indicated samples. The proteins were analysed by immuno-blotting with anti-Gwl and Gwl pThr194 antibodies. (**G**) Centrosome staining of Thr194 phosphorylated Gwl in prophase cells using co-localisation with γ-tubulin as a centrosomal marker. (**H**) Differential Thr194 phosphorylation of Gwl at spindle poles and centromeres in metaphase and anaphase cells. CenpA staining was used as a centromeric marker. Deconvolved maximum intensity projections are shown.

To test these predictions it is necessary to monitor Gwl phosphorylation during mitotic entry and exit using a phospho-specific antibody. In order to study potential Gwl activation by phosphorylation, we scanned for Cdk consensus sites following the universal DFG motif in Gwl that marks the beginning of the T-loop/activation segment. Fig. 2A shows that there is a conserved Threonine (Thr194 in human Gwl) followed by a Proline about 20 residues downstream of the DFG motif. When mutated to Alanine, only Thr194, but not Thr193 results in a significant loss of Gwl activity (Fig. 2B and C) whereas a Thr194Ser mutation does not affect Gwl kinase activity (Fig. S2A). The equivalent of human Gwl Thr194 in Xenopus is also required for kinase activity and mutants lacking this phosphorylation site are unable to reconstitute Gwl depleted egg extracts, suggesting that phosphorylation of this residue is essential for survival [22]. We therefore raised a phospho-specific pThr194 antibody to monitor Gwl phosphorylation at this residue. Fig. 2D shows that the antibody detects Flag-Gwl immuno-precipitated from nocodazole-arrested cells. We detected only weak signal in Gwl purified from asynchronous, and no signal in Gwl from Cdk1 inhibited cells, and also in a mitotic Thr194Ala mutant of Gwl. The antibody also detected Gwl specifically in mitotically enriched cells following a double Thymidine block release synchronisation (Fig. 2E and Fig. S2B). To determine, if Thr194 was directly phosphorylated by Cdk, we incubated WT and Thr194Ala Flag-Gwl that was immuno-precipitated from asynchronous human cells with recombinant cycA/Cdk2 in the presence of ATP (Fig. 2F). Addition of recombinant CycA/Cdk2 resulted in strong Thr194 phosphorylation. Applying alkaline phosphatase to the reaction significantly reduced the signal and the Thr194Ala mutant did not cross-react with the phospho-specific antibody after incubation with cycA/Cdk2. These data demonstrate that Thr194 is a Cdk site that is phosphorylated during mitosis in human cells and that the Gwl pThr194 antibody specifically cross-reacts with this phosphorylated residue.

We also determined the precise localization and timing of Gwl Thr194 phosphorylation by immunofluorescence (Fig. S2C–D). The pThr194 antibody strongly stained mitotically arrested cells and this signal was absent in most mitotic cells after Gwl siRNA depletion (Fig. S2C). There was little background signal detectable in interphase cells, but Gwl Thr194 phosphorylation occurred in the nucleus of G2 cells with separated centrosomes, increased in metaphase cells on the mitotic spindle and decreased to background levels in telophase cells (Fig. S2D). In prophase cells Gwl Thr194 phosphorylation occurred both in the nucleus and at the centrosomes (Fig. 2G). Mitotic Gwl was specifically enriched at the spindle poles and also showed distinctive foci in the metaphase plate that overlap with CenpA, suggestive of centromeric localization (Fig. 2H). This centromeric and polar phosphorylation was absent in early anaphase cells, while cytoplasmic Gwl appeared to remain phosphorylated, indicating a localised phosphatase activation in the early anaphase spindle.

### 3) Gwl Thr194 but not Ensa/ARPP19 is regulated by an OA sensitive phosphatase

**Figure 3:**
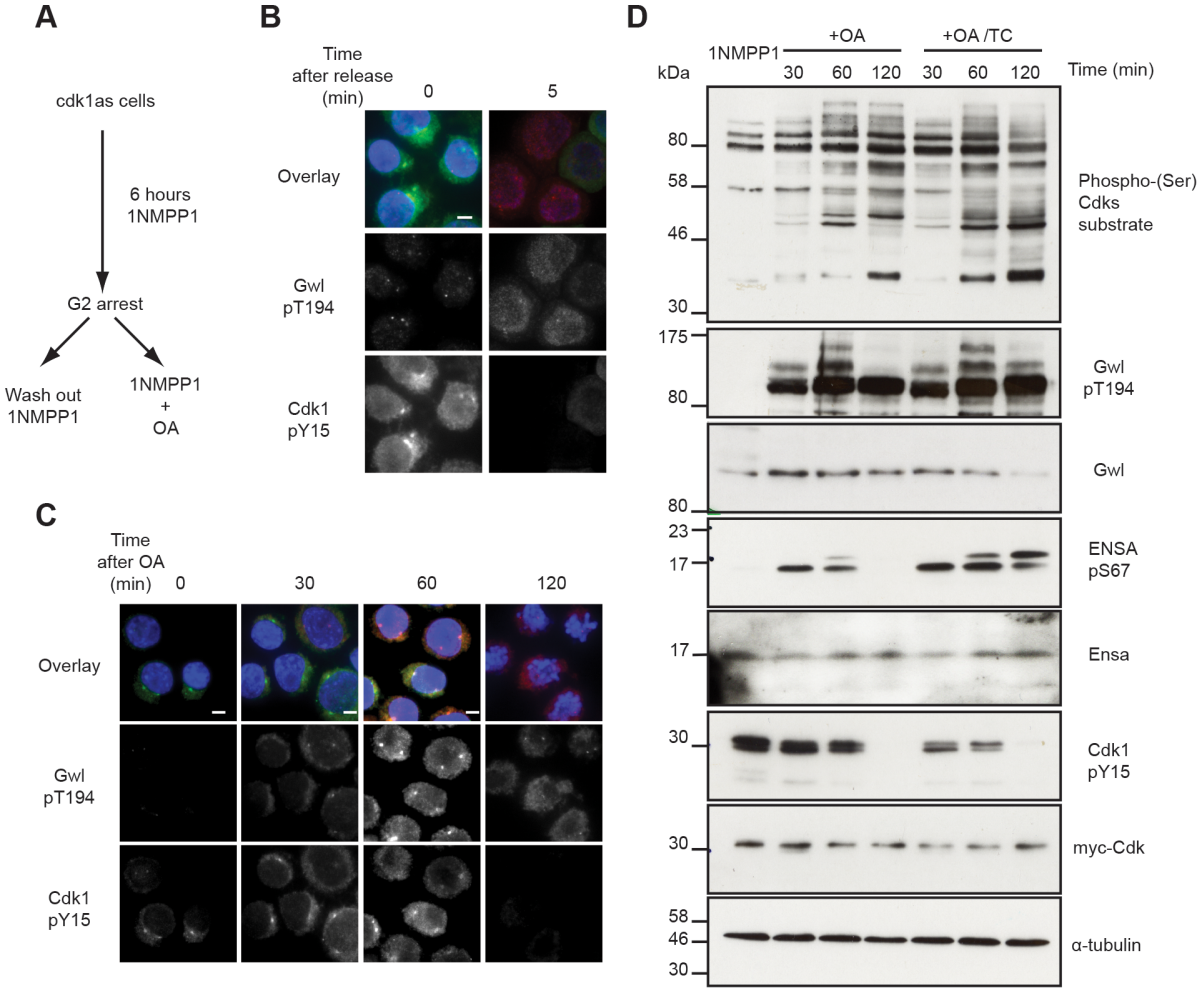
Testing Gwl and Ensa/ARPP19 phosphorylation in the G2/M switch. (**A**) Experimental protocol: DT40 cdk1as cells are blocked in G2 phase by 1NMPP1. Mitosis is triggered either by removing 1NMPP1 from the media, or by treating the cells with 1 µM OA. (**B**) Immuno-fluorescence analysis of cdk1as cells before (0) and 5 minutes after (5) release from G2 by 1NMPP1 removal using DAPI and the indicated antibodies. (**C**) Immuno-fluorescence analysis of mitotic entry in 1NMPP1 arrested cdk1as cells at indicated timepoints after treatment with 1 µM OA. (**D**) Immuno-blot analysis of 1NMPP1 treated cdk1as cell extracts taken at indicated timepoints following OA treatment, or from cells treated with both 1 µM OA and 10 µM TC.

The availability of antibodies to monitor Cdk, Gwl and Ensa/ARPP19 phosphorylation allowed us to experimentally test the mathematical models described in Fig. 1. This required a system to specifically inactivate and rapidly re-activate Cdk1. For this purpose we used the previously published cdk1as DT40 cell-line [27]. These chicken lymphocytes have the advantage of a rapid and complete re-activation of Cdk1 following removal of the ATP analogue inhibitor 1NMPP1. Thr194 was not phosphorylated in 1NMPP1 inhibited cdk1as cells, suggesting that *in vivo* Cdk1 is required for this phosphorylation. Release from Cdk1 inhibition by 1NMPP1 resulted in rapid simultaneous Cdk1 Tyr15 de-phosphorylation and Gwl Thr194 phosphorylation within 5 minutes (Fig. 3A and B). As predicted by the models presented in Fig. 1E–G, treatment of the 1NMPP1 inhibited cells with OA triggered a much slower G2/M switch and caused Cdk1 Tyr15 dephosphorylation after 60 minutes of phosphatase inhibition and a steady increase in Cdk substrate phosphorylation (Fig. 3C and D). Gwl Thr194 and Ensa/ARPP19 phosphorylation occurred rapidly within 30 minutes of OA addition (Fig. 3C and D). These data correlate with the models in Fig. 1F and G demonstrating that Gwl phosphatase is OA sensitive. The experiments also verify the prediction that Gwl phosphorylation precedes Cdk1 dephosphorylation.

Our model was built on the assumption that Ensa/ARPP19 is targeted by a constitutive phosphatase. Therefore, PP2A inhibition induced Gwl activation should result in a steady increase of phosphorylation of Ensa/ARPP19. However, Ensa/ARPP19 phosphorylation initially increased at 30 and 60 minutes after OA, but was lost at the 120 minutes timepoint, when Cdk1 was fully dephosphorylated (Fig. 3D). This prompted us to hypothesize that perhaps another OA insensitive phosphatase is activated at this late time point that can target Ensa/ARPP19, but not Gwl Thr194 phosphorylation. To test, if this activity was sensitive to other phosphatase inhibitors we repeated the experiment in the presence of the PP1 inhibitor tautomycin (TC). Fig. 3D (OA+TC) shows that this alternative inhibitor is indeed sufficient to suppress Ensa/ARPP19 dephosphorylation at the 120 minute timepoint. These data suggest that Gwl Thr194 phosphorylation is opposed by an OA sensitive phosphatase and that this phosphorylation occurs before Tyr15 dephosphorylation. Our data also suggest that Ensa/ARPP19 phosphorylation is targeted by a different phosphatase that is sensitive to TC, or a combination of OA and TC.

### 4) Dephosphorylation dynamics during mitotic exit

To gain further insight into the dephosphorylation dynamics of Gwl-Ensa/ARPP19 and Cdk substrates, we experimentally tested the mitotic exit models in Fig. 1H–J. For this purpose we arrested cells in mitosis and then triggered mitotic exit by Cdk1 inhibition. The effects of Cdk inhibitors on metaphase arrested cells are somewhat contentious [28]–[30]. To try and address this problem, we performed the mitotic exit experiment with three different Cdk inhibitors in the presence of proteasome inhibition and OA (Fig. S3A and S3B). Cdk substrate dephosphorylation was triggered by Roscovitine, Flavopiridol and RO3306 and progressed with comparable dynamics in the presence of proteasome and PP2A inhibition. However, OA appeared to cause an increase in Cdk phosphorylation activity in the metaphase arrested cells suggesting a role of OA sensitive phosphatases such as PP2A on Cdk substrate phosphorylation at this cell cycle stage. The effect of the Cdk inhibitors was not a result of off-target effects on Gwl, because none of the compounds affected Gwl *in vitro* activity (Fig. S3C).

**Figure 4:**
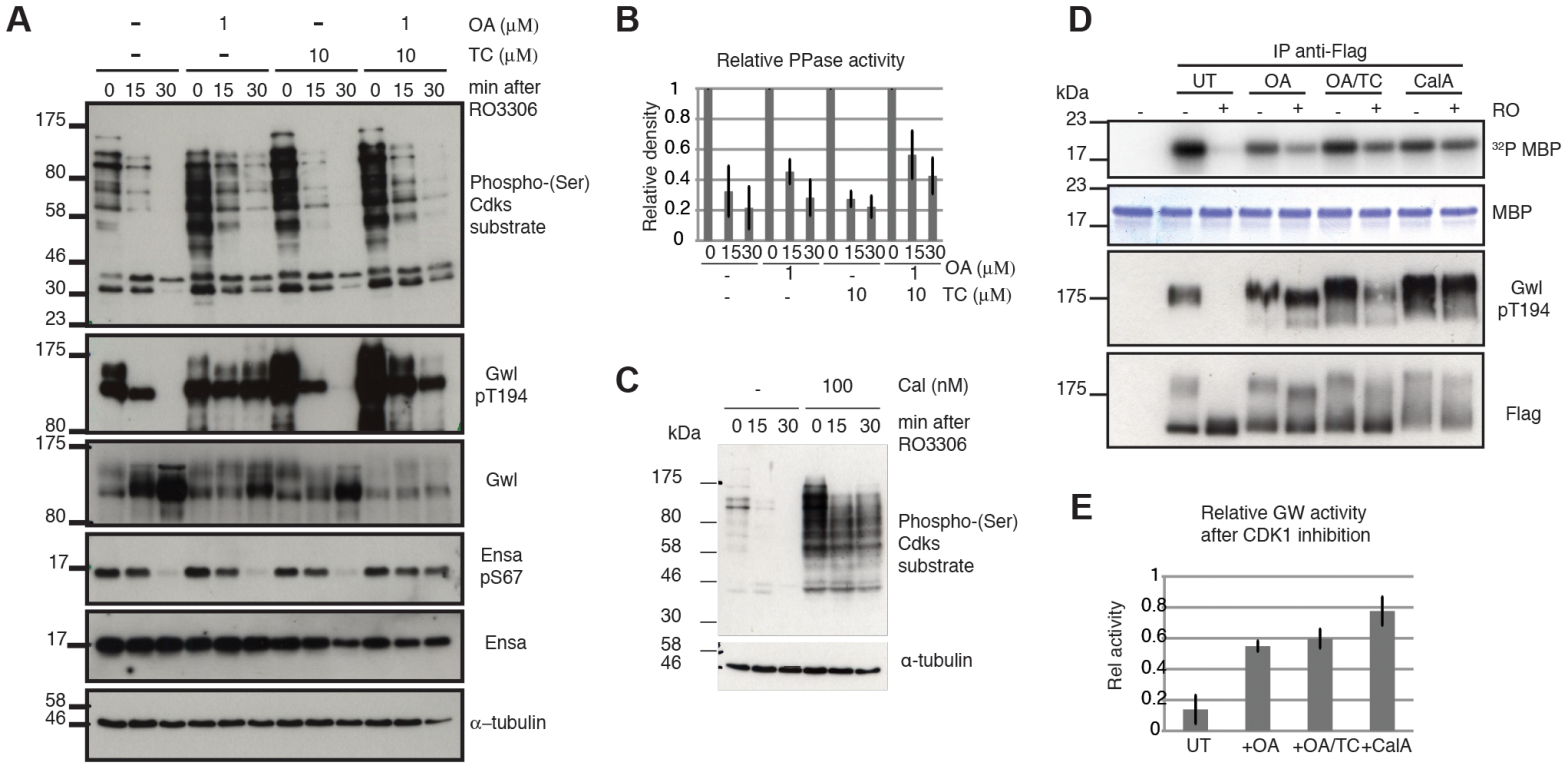
Characterizing Gwl, Ensa/ARPP19 and SP dephosphorylation during mitotic exit. (**A**) HeLa cells were synchronized in mitosis by Eg5 inhibition using 5 µM STLC and pretreated for one hour with 1 µM OA, 10 µM TC or both. Samples were taken for extraction and immunoblot analysis at indicated timepoints following treatment with 10 µM RO3306. (**B**) Quantification of relative Cdk substrate (phospho-SP) dephosphorylation in OA and TC treated cells. Error bars indicate standard deviation calculated from three independent experiments. (**C**) Immunoblot analysis of STLC arrested cells following one hour treatment with 100 nM Calyculin A (CalA). Extracts were taken at indicated times after Cdk1 inhibition by 10 µM RO3306. (**D**) MBP Kinase assays with immuno-precipitated Flag-Gwl that was transiently expressed in HeLa cells. Flag-Gwl was purified from STLC arrested cells before and 30 minutes after RO3306 treatment. Cells were pretreated for one hour with the indicated phosphatase inhibitors. (**E**) Quantification of MBP kinase activity following Cdk1 inhibition (+RO) relative to mitotic cells before Cdk1 inhibition (-RO). Error bars indicate the standard deviation calculated from three independent experiments.

For the purpose of testing the model, we chose the Cdk1 inhibitor RO3306. We blocked cells in mitosis using the Eg5 inhibitor STLC, and triggered mitotic exit by Cdk1 inhibition with RO3306. Our model predicts that Cdk1 inactivation is sufficient to trigger mitotic exit and that Gwl and Ensa/ARPP19 dephosphorylation would progress slowly, if the counteracting phosphatase is locked in a double negative feedback loop with Gwl, which is indeed what was observed. Gwl, Ensa/ARPP19 and Cdk substrate dephosphorylation all occur with a 15–30 minutes delay (Fig. 4A left most panel). We then tested the sensitivity of these dephosphorylation events to OA and TC. In accordance with our results presented in Fig. 3C & D, this experiment suggests that Gwl Thr194 dephosphorylation is OA sensitive, while Ensa/ARPP19 dephosphorylation is only inhibited in the presence of both OA and TC that inhibit both PP2A and PP1 (Fig. 4A). Accordingly, a higher dose OA concentration of 5 µM can also inhibit Ensa dephoshorylation (Fig. S3D). Both OA and TC did cause an increase in Cdk phosphorylation in the mitotic cells. However, Cdk substrate dephosphorylation did appear to proceed with unperturbed dynamics in the OA+TC treated cells following Cdk1 inhibition and was only blocked in the presence of a more global phosphatase inhibitor Calyculin A (CalA) (Fig. 4E).

We also observed that Gwl still shifts towards lower electrophoretic mobility, suggesting additional dephosphorylation of other residues, despite the presence of OA and TC and the block in Thr194 dephosphorylation (Fig. 4A). This suggests that other phosphatases may also contribute to Gwl dephosphorylation at residues different from Thr194. This notion prompted us to analyse the effect of phosphatase inhibitors on Gwl activity. For this purpose we expressed Flag-Gwl in HeLa cells synchronised in mitosis by STLC, and measured the *in vitro* kinase activity of immuno-precipitated Gwl before and after Cdk1 inhibition in the presence of different phosphatase inhibitors. Cdk1 inactivation caused a reduction to 10% of mitotic Gwl activity as well as Thr194 dephosphorylation and disappearance of the Gwl bandshifts (Fig. 4C and D). After OA addition, Gwl Thr194 dephosphorylation remained unchanged, and the protein remained active to a level of 60% relative to the mitotic control. Surprisingly, addition of TC caused a small increase in Thr194 dephosphorylation, but did not significantly affect Gwl activity. In both cases (OA and OA+TC) the high bandshift Gwl bands, were only gradually shifted down compared to the untreated control. Calyculin A led to a much more significant change in electrophoretic mobility of Gwl and blocked the inactivation of Gwl following Cdk1 inhibition to give about 80% activity, relative to the mitotic control. This suggests that Thr194 phosphorylation, as well as the bulk activity of Gwl, is counteracted by an OA sensitive phosphatase. However, other phosphorylation events in the protein are controlled by OA resistant phosphatases that have an additional but smaller impact on kinase activity.

### 5) Differential requirement for PP2A/B55 and Fcp1 to dephosphorylate Gwl and Ensa/ARPP19

**Figure 5:**
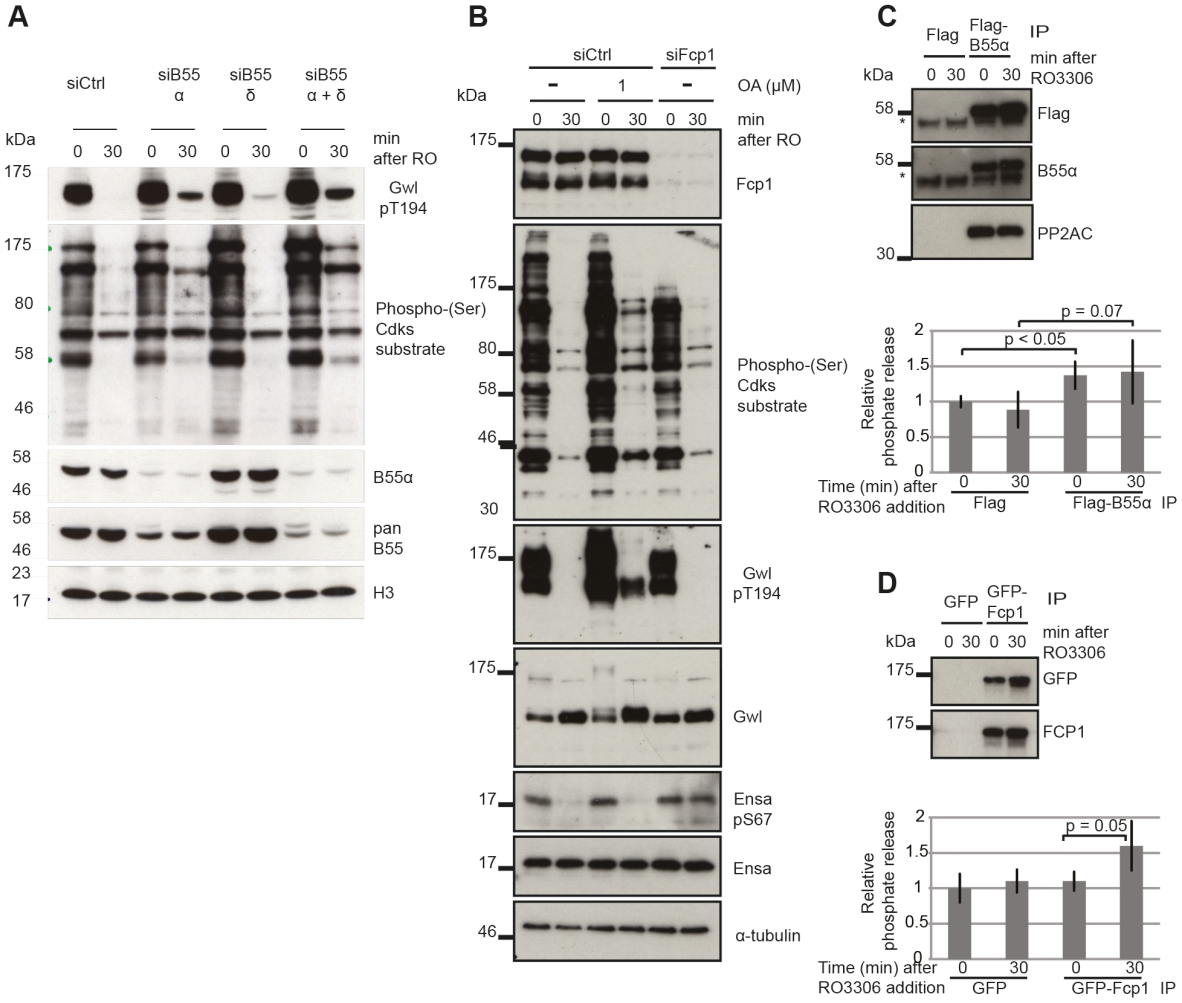
Identifying the phosphatases required for Gwl and Ensa/ARPP19. (A) Dephosphorylation of Gwl and SP sites in B55 depleted cells. HeLa cells were transfected with combinations of B55α and δ siRNAs and synchronized in mitosis with STLC as above (see Materials & Methods). Cell extracts were sampled before and 30 minutes after RO3306 treatment and analyzed by immuno-blotting with indicated antibodies. (B) Dephosphorylation of Gwl, Ensa/Arpp19 and SP sites in Fcp1 depleted cells. HeLa cells were transfected with Fcp1 siRNA and synchronized in mitosis with STLC (see Materials & Methods). Cell extracts were sampled before and 30 minutes after RO3306 treatment and analyzed by immune-blotting with indicated antibodies. (C) Gwl phosphatase assay with immuno-precipitated Flag-B55α. Purified his-Gwl was phosphorylated by Cdk2/cycA and γ^32^P ATP. Cdk2/cycA was removed from the reaction by further purification with Ni-Agarose beads and radiolabeled phospho-Gwl was incubated with immunoprecipitated B55α. The presence of the PP2A/B55α complex in the immune-precipitate was confirmed by immuno-blotting. Phosphatase activity was measured by scintillation counting of released [^32^]P phosphate. (D) Similar phosphatase assay as in (C) with recombinant *in vitro* phosphorylated Ensa and immuno-precipitated GFP-Fcp1.

PP2A/B55 itself is a good candidate for the Gwl Thr194 phosphatase, but the experiments with OA do not allow discriminating between different PP2A complexes and other OA sensitive phosphatases. This is suggested by the slow dephosphorylation following Cdk1 inactivation (Fig. 4A) fitting to a model of double negative feedback between Gwl and the Gwl phosphatase (Fig. 1J). To test this hypothesis we performed siRNA depletion of B55α and δ subunits and monitored Gwl dephosphorylation following Cdk1 inactivation of STLC synchronized mitotic cells (Fig. 5A). Depletion of B55α and δ had a synergistic effect and significantly blocked Gwl Thr194 dephosphorylation. This demonstrates that both PP2A/B55α and δ contribute to this event. Similar to the OA experiments B55α and δ depletion did cause an increase in metaphase Cdk substrate phosphorylation but did not affect the relative amount of Cdk1 substrate dephosphorylation following Cdk1 inhibition.

The results presented in Fig. 3 and 4 demonstrate that the identity of the Ensa/ARPP19 phosphatase is different from the ones targeting Gwl and other Cdk substrates. However, these data do not allow us to conclude on the nature of this phosphatase other than its sensitivity to a combination of OA and TC. The RNA polymerase II C-terminal tail domain phosphatase Fcp1 has recently been shown to play an important role during mitotic exit [31], [32]. We hypothesised that Fcp1 may be required for Ensa/ARPP19 dephosphorylation and tested this idea using Fcp1 siRNA. Fig. 5B shows that depletion of Fcp1 had no effect on Gwl Thr194 and Cdk substrate dephosphorylation following Cdk1 inactivation, but blocked dephosphorylation of Ser67 in Ensa/ARPP19.

To confirm that PP2A/B55 and Fcp1 can directly dephosphorylate Gwl and Ensa, we set up *in vitro* phosphatase assays using recombinant substrates [12] (see Materials and Methods). We purified transiently expressed Flag-B55α and GFP-Fcp1 from nocodazole treated mitotic Hek293T cells before or after Cdk1 inhibition by Flag and GFP immuno-precipitation (Fig. 5C and D). In the case of Gwl we observed a significant increase in phosphatase activity with B55α purified from mitotic cells before, or after Cdk1 inhibition (Fig. 5C). This could mean that PP2A/B55 is already active in a mitotic arrest, or that we activated it during the purification process. GFP-Fcp1, on the other hand, appeared to dephosphorylate Ensa only when purified from mitotic extracts after Cdk1 inhibition (Fig. 5D), suggesting a more stable (potentially post-translational) regulation of this phosphatase during mitotic exit. We also tested the sensitivity profile of Fcp1 against phosphatase inhibitors to determine whether OA and TC synergistically inactivate it, as suggested by the results in Fig. 4A. *In vitro*, both 1 µM OA and 10 µM TC were sufficient to inhibit this Ensa phosphatase (Figure S3E). It is difficult to relate the *in vitro* and *in vivo* concentrations of the phosphatase inhibitors, because of transport into and out of the cell and competitive binding between different phosphatases. Most likely, the combination of OA and TC has a synergistic effect in inhibiting Fcp1, but we cannot rule out indirect effects from inhibition of other phosphatases by combining OA and TC.

## Discussion

**Figure 6:**
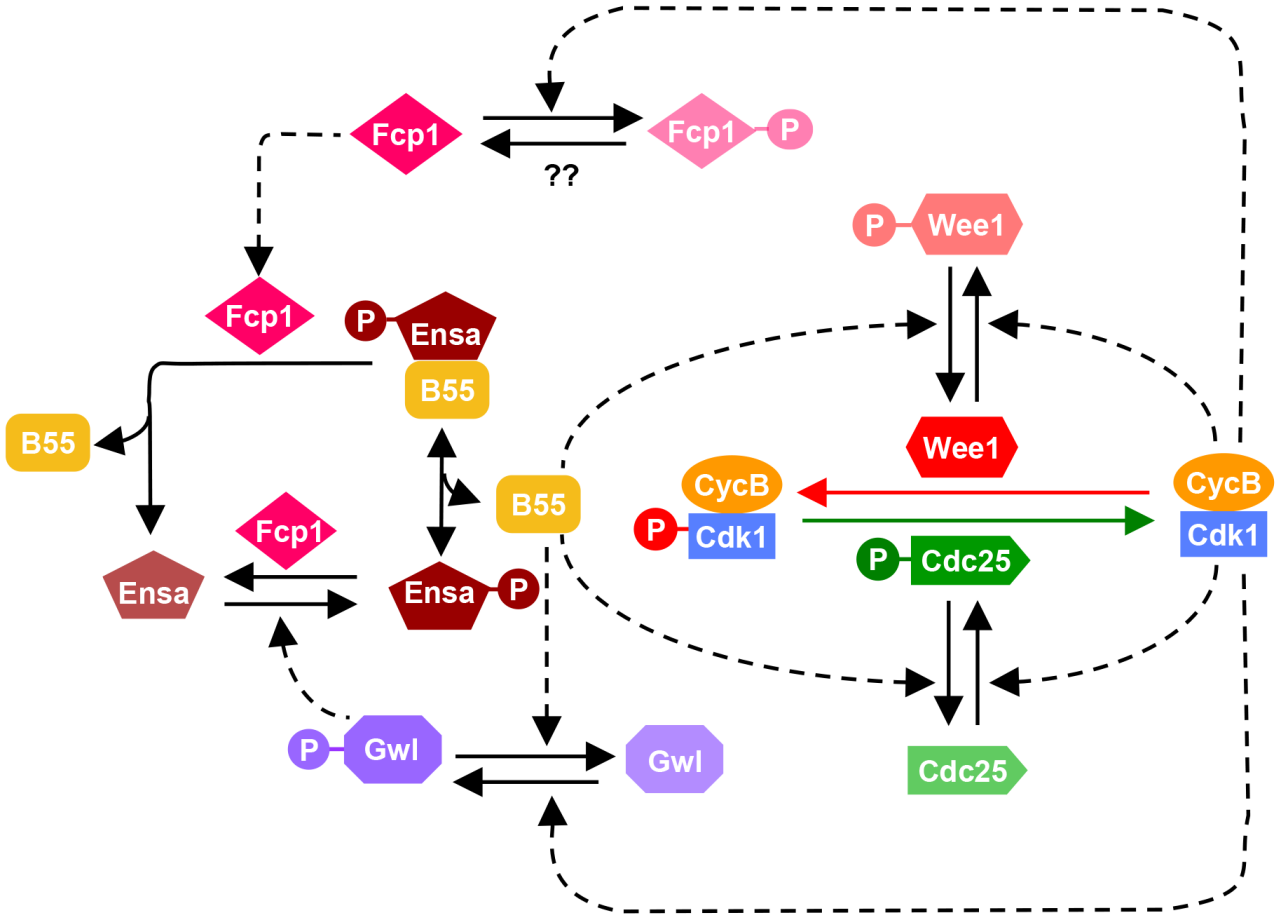
Revised model of the Cdk1 activation loop. The regulation of Gwl dephosphorylation by PP2A/B55 and Ensa/ARPP19 dephosphorylation by Fcp1 are incorporated into the previous network shown on Fig. 1A. Since Fcp1 is inhibited in Cdk1 dependent manner, Ensa is regulated by a coherent feed-forward loop as well: Cdk1 both activates the phosphorylation (via Gwl) and inhibits the dephosphorylation (via Fcp1) of Ensa.

The results presented here suggest a complex phosphatase network counteracting the Cdk1 activation loop. These data allow us to update the model of the mammalian mitotic switch by including the new information on PP2A/B55 and Fcp1 (Fig. 6). The inclusion of an additional layer of phosphatase regulation does not alter the fundamental characteristic of the mitotic switch model, but it influences the temporal dynamics of mitotic entry and exit. Our mathematical modeling together with experiments indicates that mutual antagonism between Gwl and PP2A/B55 accelerates both mitotic entry and exit with synthesis and degradation of CycB, respectively. Our data also indicate that while PP2A is principally responsible for the inactivation step of Gwl, phosphatases other than PP2A regulate additional phosphorylation sites and also contribute to Gwl inactivation (see Fig. 4C and D). We have also identified Fcp1 as the phosphatase counteracting the Gwl phosphorylation site in Ensa/ARPP19. This implicates that Fcp1 is yet another intrinsic element of the mitotic switch and helps explain the observation that this phosphatase is required for mitotic exit [32]. Fcp1 appears to be subjected to further regulation by Cdk1 and activated during mitotic exit. These novel elements of the mitotic switch remain to be investigated.

Strikingly neither PP2A, PP1 nor Fcp1 appear to be sufficient to dephosphorylate the bulk of mitotic Cdk1 substrates, if the Gwl feedback loop is bypassed by Cdk1 inhibition. Dephosphorylation of the majority of Cdk substrates proceeds despite the presence of inhibitors (Fig. 4A), depletion of B55 subunits (Fig. 5A) and Fcp1 (Fig. 5B) and persistent activation of Gwl and Ensa (Fig. 4A and 5B), which additionally inhibit PP2A/B55. These results suggest that PP2A/B55 is not the only mitotic exit phosphatase that removes the bulk of mitotic phosphorylation. This notion is also supported by experiments in Xenopus egg extracts, in which Cdk1 inhibition by p27 can trigger mitotic dephosphorylation even in the absence of B55δ [12]. This is further supported by the observation that B55 depleted cells do not show a prolonged arrest in mitosis, as would be expected in the absence of the major Cdk1 antagonizing phosphatase [26]. Thus, the nature of the phosphatases that directly counteracts Cdk1 during the mitotic exit in mammalian cells remains elusive. In yeast Cdc14 covers this role, but single deletions of the mammalian equivalents Cdc14 A and B do not have the expected mitotic phenotypes [33], [34]. However, a double deletion of both orthologues has, to our knowledge, not been reported. Further experiments will be necessary to identify the mammalian version of this crucial mitotic exit phosphatase, and to analyse how it is integrated in the mitotic entry and exit switches.

## Materials and Methods

Entry into mitosis is triggered by the activation of Cdk1/CycB. The network that controls Cdk1/CycB activity involves regulation of inhibitory kinase, Wee1 and activatory phosphatase, Cdc25. In interphase, Wee1 dependent phosphorylation keeps Cdk1 inactive while Cdc25 dependent dephosphorylation activates Cdk1 and promotes entry into mitosis. Both Wee1 and Cdc25 are subjected to Cdk1/CycB dependent phosphorylations which lead to the inactivation of Wee1 and activation of Cdc25. As a consequence, Cdk1 activity is controlled by feedback loops involving Wee1 (Cdk1—|Wee1—|Cdk1) and Cdc25 (Cdk1→Cdc25→Cdk1). Cdk1 subunit is in excess compared to its activating partner Cyclin B. Therefore, it is assumed that all Cyclin B is in the complex such that total CyclinB (CycBT) is sum of active Cdk1/CycB and inactive Cdk1/CycB dimers.

The network also includes the regulation of Cdk1/CycB counteracting phosphatase, PP2A/B55. We consider that the dephosphorylation of Wee1 and Cdc25 is controlled by PP2A/B55. Cdk1 indirectly inhibits PP2A/B55 via Gwl-Ensa/ARPP19 pathway. Thus, Cdk1 promote Wee1 and Cdc25 phosphorylations by directly phosphorylating them and by indirectly inhibiting their phosphatase forming coherent feed-forward loops. Gwl-Ensa/ARPP19 pathway involves Cdk1/CycB dependent phosphorylation of Gwl (Gwl) which in turn phosphorylate the phosphatase inhibitor Ensa/ARPP19. The phosphorylated Ensa/ARPP19 binds directly to PP2A/B55 to form an inhibitory complex. The phosphatases that dephosphorylate Gwl and Ensa/ARPP19 are unknown. Therefore, we consider three different scenarios. Gwl is dephosphorylated by (A) OA insensitive phosphatase (B) OA sensitive phosphatase (PP2A) and (c) PP2A/B55. We also assume that ENSAPt (sum of phosphorylated forms of Ensa/ARPP19) is dephosphoryated by OA insensitive phosphatase. We predict using the model the effect of different phosphatases on both the steady state of the system and dynamics of chemical inhibitor induced mitotic entry and exit.

The temporal dynamics of each component in the network is described by non-linear ordinary differential equation (ODE). All the individual biochemical reactions are approximated by mass action kinetics. The set of ODEs are integrated numerically using stiff solver in XPPAUT, a freely available program from G. Bard Ermentrout, University of Pittsburgh, PA, USA; http://www.math.pitt.edu/~bard/xpp/xpponw95.html).

One parameter bifurcation diagrams (Suppl. Fig. S1) were computed using the program AUTO. Bifurcation diagram is used to illustrate how steady state of the dynamical system changes as a function of control parameter. We compute the diagram with respect to CycBT as a parameter since its level is unaffected by the dynamics of the system. We present the XPPAUT code of model that can be used to re-produce all the simulations (Fig. 1 and Fig. S1) in the manuscript. The comparative analysis of the three models is carried out by using the same set of parameter values except for the changes corresponding to Gwl phosphatase (see below). The total concentrations of the proteins are assumed to be equal to one except for PP2A (PP2t = 0.5) whose concentration is kept below its stoichiometric inhibitor, ENSA concentration. The choice of kinetic parameter values is based on the criteria that G2/M transition should exhibit bistable characteristic [4] [6]. Two parameter bifurcation diagrams were used to study the dependence of bistability on kinetic parameter values. We observed bistable behaviour over a wide range of kinetic parameters values (kagwl>1; kigwl′ = 0–10; kaensa>1; kiensa = 0–2.5) and within this range the dynamical characteristics of the three models as shown in Figure 1B–J are not affected. These characteristics include rapid Tyr15 dephosphorylation (in all the three models) during mitotic entry with Cdk1 inhibition and release in comparison with the Cdk1 inhibition + OA and delay in Gwl inactivation during mitotic exit only when Gwl is dephosphorylated by PP2A/B55. The inclusion of PP2A/B55 dependent regulation of Gwl also makes the system bistable over wider range of total Cyclin B levels, because of extra double–negative feedback loop between Gwl and PP2A/B55.

The parameter values in XPPAUT code correspond to the situation of Gwl dephosphorylation by PP2A/B55 and the other two models can be simulated with following changes: Gwl dephosphorylation by OA insensitive phosphatase: kigwl′ = 2; kigwl = 0.

Gwl dephosphorylation by OA sensitive phosphatase (PP2A): kigwl″ = 2; kigwl = 0.

The chemical inhibition of Cdk1 and PP2A are modelled as a reversible inhibitor binding reaction. The mitotic entry simulation in the presence of inhibitor is done with parameter value RO = 25 (for Cdk1 inhibition), OA = 100 (for PP2A inhibition).The mitotic exit simulation in the presence of inhibitor is done with parameter value RO = 100 (for Cdk1 inhibition).

### XPPAUT file containing ODEs, parameter values and initial conditions

# Initial conditions

init MPF = 0, Cdc25 = 0, Wee1 = 1, Gwl = 0, ENSAPt = 0, PP2 = 0.5

# Use the following initial conditions for mitotic exit

# MPF = 0.96, Cdc25 = 0.97, Wee1 = 0.03, Gwl = 0.9, ENSAPt = 0.75, PP2 = 0.027

#Differential equations

MPF′ = k25*(CycT-MPF) - kwee*MPF

Cdc25′ = Va25*MPFa*(Cdc25T-Cdc25) - Vi25*PP2a*Cdc25

Wee1′ = Vawee*PP2a*(Wee1T-Wee1) - Viwee*MPFa*Wee1

Gwl′ = kagwl*MPFa*(GwlT-Gwl) - (kigwl′+ kigwl″*PP2T/(1+OA)+ kigwl*PP2a)*Gwl

ENSAPt′ = kaensa*Gwl*(ENSAT - ENSAPt) - kiensa*ENSAPt

PP2′ = -kas*(ENSAPt - (PP2T-PP2))*PP2 + (kdis + kiensa)*(PP2T-PP2)

MPFa = MPF/(1+RO)

PP2a = PP2/(1+OA)

aux preMPF = CycT-MPF

aux PP2a = PP2/(1+OA)

aux MPFa = MPF/(1+RO)

k25 = k25′*(Cdc25T-Cdc25) + k25″*Cdc25

kwee = kwee′*(Wee1T -Wee1) + kwee″*Wee1

#parameters

par CycT = 1, Va25 = 2, Vi25 = 2, Vawee = 2, Viwee = 2

par kagwl = 10, kigwl′ = 0.02, kigwl″ = 0, kigwl = 2, kaensa = 2, kiensa = 0.6

par kas = 100, kdis = 1

par Cdc25T = 1, Wee1T = 1, GwlT = 1, ENSAT = 1, PP2T = 0.5,

par k25′ = 0.01, k25″ = 1, kwee′ = 0.01, kwee″ = 1

par RO = 0, OA = 0

@ total = 30,dt = 0.5,meth = STIFF

@ xlo = 0,xhi = 30,ylo = 0,yhi = 1

@ NPLOT = 6, yp1 = preMPF, yp2 = Cdc25, yp3 = ENSAPt, yp4 = PP2a, yp5 = Gwl

@ NTST = 15,NMAX = 20000,NPR = 1000,DS = 0.02

@ DSMAX = 0.05,DSMIN = 0.001,PARMIN = -1,PARMAX = 1

@ AUTOXMIN = 0,AUTOXMAX = 1,AUTOYMIN = 0,AUTOYMAX = 1

done

### Cloning and mutagenesis

Gwl cDNA was cloned by RT-PCR from mRNA isolated from HeLa cells using RNeasy mini kit (Quiagen). Reverse-transcriptase PCRs (RT PCRs) were performed using the SuperScript III One-Step RT-PCR System from Invitrogen. Gwl cDNA was amplified from HeLa cell messenger RNA (mRNA) (using primers 5′GGG GAC AAG TTT GTA CAA AAA AGC AGG CTT AAT GGA TCC CAC CGC GGG AAG c3′; 5′GGG GAC CAC TTT GTA CAA GAA AGC TGG GTC CTA CAG ACT AAA TCC AGA TAC GG3′) and cloned into the pCR II-TOPO cloning vector using the TOPO-TA Cloning Kit from Invitrogen. In all cases the manufacturer's recommended protocols were followed and the presence of the expected DNA sequences confirmed by restriction digestion and sequencing. The cDNA was then cloned into an N-terminal Flag tag mammalian expression destination vector (a gift from Dr. Stephan Geley, University of Innsbruck, Austria). Site directed mutagenesis was carried out using the QuickChange XL Site-Directed Mutagenesis Kit from Stratagene (California, USA) following the manufacturer's protocol. Human B55α was cloned by RT-PCR as above using the primers 5′GGG GAC AAG TTT GTA CAA AAA AGC AGG CTT A ATG GCA GGA GCT GGA GGA GG3′ and 5′GGG GAC CAC TTT GTA CAA GAA AGC TGG GTC CTA ATT CAC TTT GTC TTG AA3′ cloned into a Gateway Flag-tag expression vector. Fcp1 cDNA was synthesized by Genscript and cloned into the EGFP-C1 vector via Bgl2 and EcoR1 sites. Human Ensa was cloned by RT-PCR using the primers 5′ GGG G ACA AGT TTG TAC AAA AAA GCA GGC TTA ATG TCC CAG AAA CAA GAA GA 3′ and 5′ GGG GAC CAC TTT GTA CAA GAA AGC TGG GTC TCA TTC AAC TTG GCC ACC CG3′ and further cloned into a his-tag Gateway bacterial expression vector (a gift from Dr. Stephan Geley, University of Innsbruck, Austria).

### Antibodies

Rabbit phospho-(Ser) CDKs substrate antibody was purchased from Cell Signaling. Peptides and polyclonal rabbit Phospho-Thr194 Gwl were generated and purified by Eurogentec, Belgium. Peptides and polyclonal rabbit phospho-Ser67 Ensa/ARPP19 was generated by Generon and we also obtained a phosphor-specific P-Ser67 Ensa antibody from Dr. Satoru Mochida (Kumamoto University, Japan). To make phospho-specific monoclonal antibody (mAB) to phospho threonine and tyrosine on cdk1, a phospho-peptide EKIGEGpTpYGVVYKGC was coupled to KLH and injected into Balb/c mice. Hybridoma cells produced from the spleen cells from a hyperimmune mouse were fused with Sp/0 myeloma cells using standard procedures. Positive clones were identified using immunoadsorbent assays and immunoblotting of Xenopus egg extracts. mAb CP3.2 gave the strongest signal in these assays.; α -tubulin (clone DM1A), myc (clone 9E10) and CENPA (clone 3–19) mouse monoclonal antibodies were purchased from Abcam (Cambridge, UK), PP2A/B55α (clone 2G9) and PP2A/B55 (clone D-10) mouse monoclonal antibodies from Santa Cruz Biotechnology (Heidelberg, Germany),PP2AC from Sigma (SAB4200266), histone H3 mouse monoclonal antibody (clone 6.6.2) from Millipore (Watford, UK) and mouse monoclonal anti-MASTL from Sigma (HPA027175). HRP-conjugated and polyclonal goat anti-rabbit or mouse antibodies were from Dakocytomation (UK). Secondary antibodies for immunofluorescence were from Invitrogen.

### Cell culture

HeLa cells were cultured in Dulbecco's modified Eagle Medium (DMEM) supplemented with 10% FBS, 2 mM L-glutamine, 100 U/mL penicillin and 0.1 mg/mL streptomycin in a 37°C, 5% CO_2_ incubator. Chicken DT40 cells including cdk1as cells were cultured as previously described (24).

### G2/M transition experiments

Cdk1as cells were synchronized in G2 with 10 µM 1NMPP1 for 6 h, then were released in mitosis after 1NMPP1 wash out and collected 5 min later for immunofluorescence assays. Alternatively, G2 cells were incubated with 1 µM of OA or/and 10 µM TC for indicated times and harvested for immuno-fluorescence and immuno-blotting analysis.

### Mitotic exit experiments

HeLa cells were synchronized with 5 µM of S-trityl-L-cysteine (STLC; Tocris, Bristol, UK) for ∼18 h, then were harvested by mitotic shake-off. The cells were then incubated in STLC-containing media supplemented with 1 µM of okadaic acid (OA; Calbiochem, Merck Chemicals, Nottingham, UK) or/and 10 µM of tautomycetin (TC; Tocris), or 100 nM of calyculin A (Calbiochem) for 1 h. Next, Cdk1 activity was inhibited with 10 µM of RO3306 (Calbiochem) for 0, 15 or 30 min and the cells were collected for immunoblotting analysis.

To knock-down PP2A/B55α and δ subunits, 40 nM of each siRNA B55 (5′-GCAGAUGAUUUGCGGAUUA-3′ for B55α and 5′-CAUCCAUAUCCGAUGUAAA-3′ for B55δ, Qiagen, Manchester, UK) were reverse transfected using Lipofectamine RNAiMAX reagent (Invitrogen) two consecutive days according to the provider's instructions. The cells were treated with STLC for mitotic synchronization 48 h after the 2^nd^ transfection and mitotic exit experiment was carried out the following day.

### Immunoblotting

Cells were lysed in 40 µL of EBC buffer (50 mM Tris pH 7.5, 120 mM NaCl, 0.5% NP40, 1 mM EDTA, 1 mM DTT, Protease and Phosphatase inhibitors (Complete and PhosStop; Roche Diagnostics, West Sussex, U.K)) and 10 µl 5× sample buffer (0.01% bromophenol blue, 62.5 mM Tris-HCl pH 6.8, 7% SDS, 20% sucrose and 5% β-mercaptoethanol). The samples were sonicated then boiled at 95°C for 5 min. Samples were then analysed by western blotting and the signal was using Immobilon Western Chemiluminescent HRP substrate (Millipore). Image J was used to quantify the intensity of phospho-(Ser) CDKs substrate signal. α-tubulin was used to normalize the samples.

### Immuno-precipitation and kinase assay

HeLa cells were transfected with 10 µg of Flag-Gwl plasmid (using GeneJuice transfection reagent (Novagen, Merck Chemicals) following the manufacture's guidelines. Hek293T cells were transfected using CaCl_2_. 24 h later, 5 µM of STLC, 75 ng/mL Nocodazole or 10 µM RO3306 was added to the media and incubated for 16–18 h. Indicated cells were then treated with 1 µM of OA or/and 10 µM of TC or 100 nM of calyculin A for 1 h. Mitotic exit was induced by adding 10 µM of RO3306 for 30 min. Cells were lysed on ice for 20 min in IP buffer (20 mM TrisHCl ph 7.5, 137 mM NaCl, 10% glycerol, 0.5% NP-40, 2 mM EDTA, Protease and Phosphatase inhibitors (Complete and PhosStop)) supplemented with the appropriate phosphatase inhibitors (OA and/or TC or Calyculin A). Protein samples were clarified by centrifugation at 13 000 rpm for 30 min at 4°C. Flag-Gwl protein was captured on anti-flag M2 magnetic beads (Sigma) for at least 2 h at 4°C. After 2 washes with IP buffer and 2 with kinase buffer (50 mM MOPS pH 7.5, 5 mM MgCl_2_, 0.4 mM EDTA and 0.4 mM EGTA), the beads were incubated with 10 µg of myelin basic protein (MBP, Millipore) diluted in kinase buffer supplemented with 25 µM of ATP and 0.003 MBq of *γ-* ^32^P*-*ATP for 20 min *at* 37°C. The reaction was stopped by adding the sample buffer and was analysed by western blot and autoradiography. Recombinant Cdk2/cycA was a gift from Dr. Julian Gannon (CRUK Clare Hall, UK).

### Immuno-fluorescence

HeLa cells were grown on coverslip, then were fixed with 3.7% of formaldehyde (Sigma-Aldrich, Dorset, UK) in PBS for 10 min (Fig. 2B) or mitotic cells were collected and were spun onto slides at 500 r.p.m. for 5 min then fixed (Fig. 2C). DT-40 cells were cyotspun at 1000 r.p.m. for 3 min then fixed. After several PBS washes, cells were permeabilized with 0.1% NP-40 in PBS fro 10–20 min then stained with indicated antibodies and mounted with Prolong Gold DAPI solution (Invitrogen, Paisley, UK).

Images were acquired on DeltaVision microscope equipped with a UPLS Apo, N.A. 1.40, 100× oil immersion objective (Olympus), standard filter sets (Excitation 360/40; 490/20; 555/28; Emission 457/50; 528/38; 617/40) and a CoolSNAP_HQ2 camera (Photometrics). Images were exported to Omero software (Version 4.4.4). Deconvolution were performed using SVI Huygens Professional Deconvolution Software (Version 3.5) and images were converted into Adobe Photoshop files.

### Phosphatase assay

Recombinant His-tagged Ensa was purified using Ni-NTA agarose (Qiagen) from BL21 E.coli following the manufacturer's protocol. 1 mg of purified protein was then phosphorylated for 2 h at 37°C by recombinant engineered Gwl kinase (Hochegger lab, unpublished results) in kinase buffer (20 mM Hepes-HCl pH 7.8, 10 mM MgCl_2_, 15 mM KCl, 1 mM EGTA, 5 mM NaF, 20 mM β-glycerophosphate, 1 mM DTT and 1.85 MBq γ-^32^P-ATP (PerkinElmer)). To eliminate unincorporated ATP and the kinase, the phosphor-Ensa protein was captured on Ni-NTA beads. After elution, the phospho substrate was concentrated by spinning on ultrafiltration columns (Vivaspin 500, 3 000 MWCO, Fisher) in buffer containing 20 mM TrisHCl pH 7.5, 150 mM NaCl and 0.01% Tween 20. Hek293 cells were transfected with GFP-FCP1 using a calcium phosphate transfection protocol. 24 h later, cells were treated with 100 ng/mL of nocodazole for 16–18 h and mitotic cells were incubated in DMSO or 10 µM of RO3306 for 30 min at 37°C. GFP-FCP1 was then immunoprecipitated from cell extracts using magnetic GFP-Trap_M beads (Chromotek, Germany) according to the provider's instructions. After washes, beads were resuspended in phosphatase buffer (20 mM HepesNaOH pH 7.9, 10 mM MgCl_2_, 20 mM KCl, 10% glycerol, 1 mM DTT and, 0.2 mM PMSF). Phospho-Ensa was added to the mixture and samples were incubated for 90 min at 30°C. The reaction was stopped by TCA protein precipitation then the samples were treated as described in [12]. The radioactivity was measured with a liquid-scintillation counter.

Recombinant His-tagged Gwl was produced in insect cells and purified on Ni-Agarose beads. and concentrated by spinning on ultrafiltration columns (Vivaspin 2, 10 000 MWCO) in 40% glycerol/PBS buffer. His-tagged Gwl was phosphorylated *in vitro* by the recombinant Cdk2/cyclinA complex (a gift from Tim Hunt). The kinase reaction and the purification of the phosphorylated substrate were similar to the preparation of the phosphorylated recombinant ENSA. Flag-B55α was transfected in HEK293 cells. The phosphatase assay with B55α was carried out in a similar way as FCP1 phosphatase assay except the Flag-B55α was captured on anti-flag M2 magnetic beads (Sigma) and phosphorylated his-Gwl was used as a substrate.

## Supporting Information

Figure S1 Steady state analysis of the mitotic switch. The steady state fraction of Tyr15 Cdk1 phosphorylation and phosphorylated Gwl are plotted as a function of CycB levels, assuming that Gwl is dephosphorylated by an OA-insensitive (A), an OA-sensitive phosphatase (B) or by PP2A/B55 (C). The ‘balance curves’ were calculated in the absence of any inhibitor (green curves), in the presence of Cdk1 inhibitor (red curves) and Cdk1 plus PP2A inhibitors (blue curves). Solid and dashed lines represent stable and unstable steady states, respectively. Down-regulation of the phosphatase on Wee1 and Cdc25 by Cdk1 activity is required to generate bistability in our model. This is achieved through the Cdk1-dependent activation of the Gwl-ENSA pathway that inhibits PP2A:B55 phosphatase. This explains the loss and the persistence of bistability after Cdk1 and PP2A combined inhibition (blue curves) with OA-insensitive (Fig. S1A) and OA-sensitive (Fig. S1B and S1C) Gwl phosphatase, respectively. An OA-insensitive phosphatase keeps Gwl unphosphorylated at low Cdk1 activity therefore PP2A:B55 activity becomes independent of Cdk1 activity. However, if Gwl is dephosphorylated by an OA-sensitive phosphatase (e.g. PP2A:B55 or any other PP2A), Gwl phosphorylation and thereby PP2A:B55 activity is still Cdk1 dependent, assuming not a complete inhibition by OA.
(TIF)

Figure S2 (**A**) WT and Thr194Ser Flag-Gwl were transiently expressed in 293T cells. 48 hours after transfection the cells were synchronized in mitosis by 18 hour incubation with nocodazole. Mutant and WT kinase were immuno-precipitated and assayed for kinase activity using MBP as a substrate. The kinase assays were analyzed by SDS PAGE and autoradiography. (**B**) FACS profile of thymidine release samples taken at indicated time points after thymidine washout. Cells passed through mitosis between 8 hours and 10 hours, correlating with Gwl Thr194 phosphorylation. (**C**) siRNA depletion of Gwl to determine specificity of the immuno-fluorescent signal of the phosphor Thr194 antibody. Cells were siRNA transfected with control and Gwl siRNA. 48 hours later, cells were treated with STLC to achieve mitotic arrest and stained with anti Gwl pThr194 (green), anti-αtubulin (red) and DAPI (blue). Note the mitotic cells that lost Gwl Thr194 signal in the knock-down sample. (**D**) Analysis of Gwl Thr194 phosphorylation by immunofluorescence with anti-Gwl pThr194 antibodies. DAPI staining and centrosome separation was used to identify mitotic cells at various stages.
(TIF)

Figure S3 Mitotic exit triggered by Cdk inhibition. (**A**) Cells were arrested in mitosis by STLC and pretreated with 1 µM OA and 20 µM MG132 before inactivation of Cdk1 by 50 µM Roscovitine (Ros), 5 µM Flavopiridaol (Fp) and 10 µM RO3306 (RO). Mitotic exit was scored by measuring levels of phosphorylated SP by immune-blots. (**B**) Three independent experiments as shown in (A) were quantified using Image J. Error bars indicate standard deviation in the 3 data sets. (**C**) Effect of Cdk inhibitors on Gwl activity measured by IP/kinase assays. Flag-Gwl was transfected in 293T cells. 48 hours after transfection the cells were arrested in nocodazole for 18 hours. Flag-Gwl was immuno-precipitated from mitotic cells and incubated with recombinant Ensa/ARPP19 and γ^32^P ATP. The kinase assays were analyzed by SDS PAGE and autoradiography. (**D**) Effects of 1 and 5 µM OA on mitotic exit dephosphorylation. HeLa cells were synchronized in mitosis by Eg5 inhibition using 5 µM STLC and pretreated for one hour with 1 µM and 5 µM OA. Samples were taken for extraction and immunoblot analysis at indicated timepoints following treatment with 10 µM RO3306. (**E**) Effects of OA and TC on FCP1 phosphatase activity. Ensa phosphatase were performed as described in Figure 5 and the reactions were incubated with 1 µM OA, 10 µM TC and 100 nM CalA.
(TIF)
