# Correction: PP2A/B55 and Fcp1 Regulate Greatwall and Ensa Dephosphorylation during Mitotic Exit

**DOI:** 10.1371/journal.pgen.1004259

**Published:** 2014-02-27

**Authors:** The PLOS Genetics Staff

There is a mistake in the alignment of the Drosophila Greatwall sequence in Figure 2A.

The correct Drosophila sequence is: fglskidmrrdleisdlincspnlnartpgqllsltshlsfgsekklndfgsvssgqnngmg

This sequence does not contain a conserved TTP Cdk consensus site at the residues equivalent to the human Threonine 193 and 194.

Note that there is a TP in this sequence, which could be a relevant Cdk phosphorylation site.

This requires further experimental evidence.
