# Supplementary figures and images for: PP2A/B55 and Fcp1 Regulate Greatwall and Ensa Dephosphorylation during Mitotic Exit
Source: PLoS Genet. 2014 Jan 2;10(1):e1004004. doi: 10.1371/journal.pgen.1004004 (PMC3879168; doi:10.1371/journal.pgen.1004004)

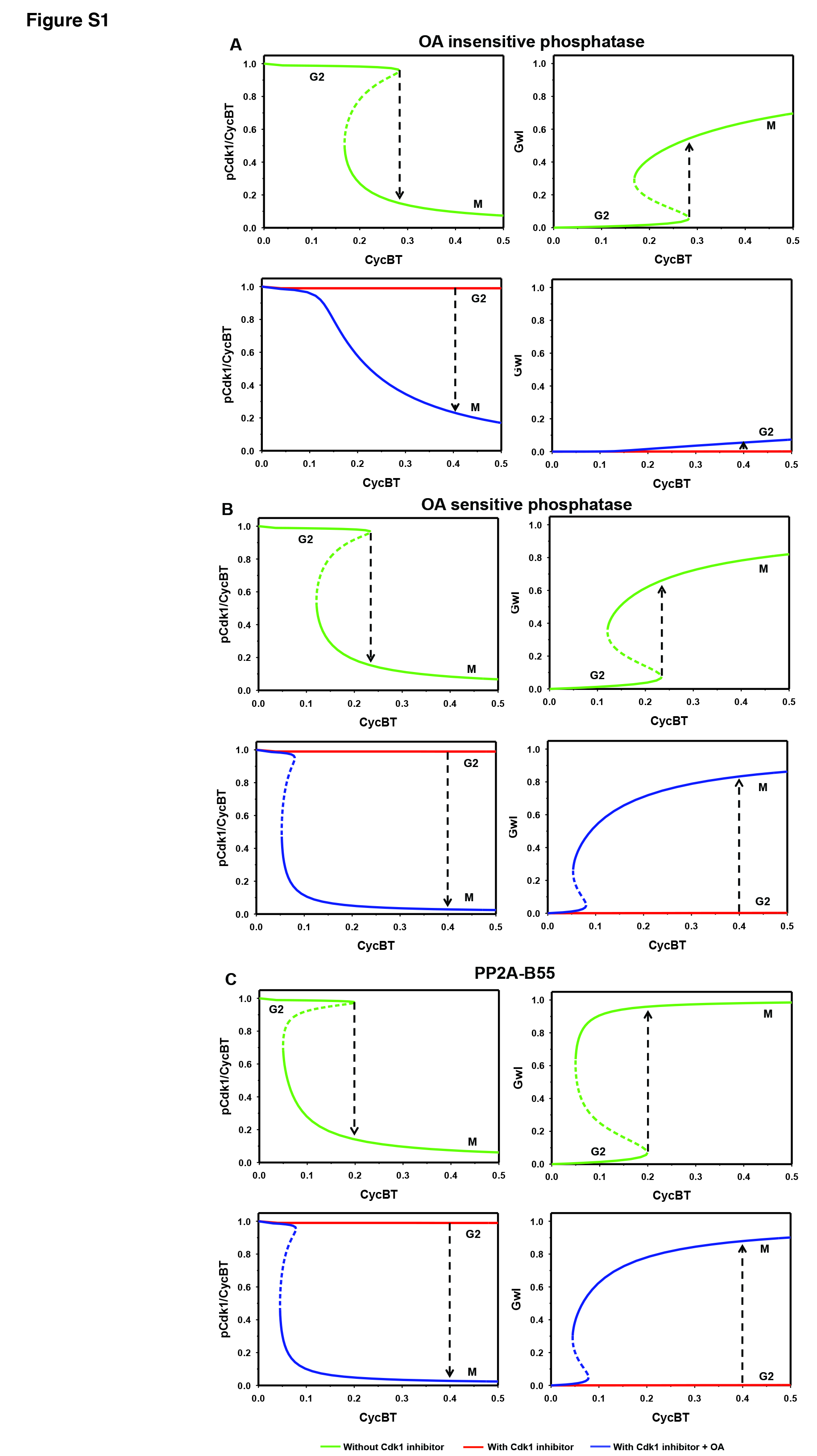

Supplement: Figure S1 — Steady state analysis of the mitotic switch. The steady state fraction of Tyr15 Cdk1 phosphorylation and phosphorylated Gwl are plotted as a function of CycB levels, assuming that Gwl is dephosphorylated by an OA-insensitive (A), an OA-sensitive phosphatase (B) or by PP2A/B55 (C). The ‘balance curves’ were calculated in the absence of any inhibitor (green curves), in the presence of Cdk1 inhibitor (red curves) and Cdk1 plus PP2A inhibitors (blue curves). Solid and dashed lines represent stable and unstable steady states, respectively. Down-regulation of the phosphatase on Wee1 and Cdc25 by Cdk1 activity is required to generate bistability in our model. This is achieved through the Cdk1-dependent activation of the Gwl-ENSA pathway that inhibits PP2A:B55 phosphatase. This explains the loss and the persistence of bistability after Cdk1 and PP2A combined inhibition (blue curves) with OA-insensitive (Fig. S1A) and OA-sensitive (Fig. S1B and S1C) Gwl phosphatase, respectively. An OA-insensitive phosphatase keeps Gwl unphosphorylated at low Cdk1 activity therefore PP2A:B55 activity becomes independent of Cdk1 activity. However, if Gwl is dephosphorylated by an OA-sensitive phosphatase (e.g. PP2A:B55 or any other PP2A), Gwl phosphorylation and thereby PP2A:B55 activity is still Cdk1 dependent, assuming not a complete inhibition by OA. (TIF) [file pgen.1004004.s001.tif]

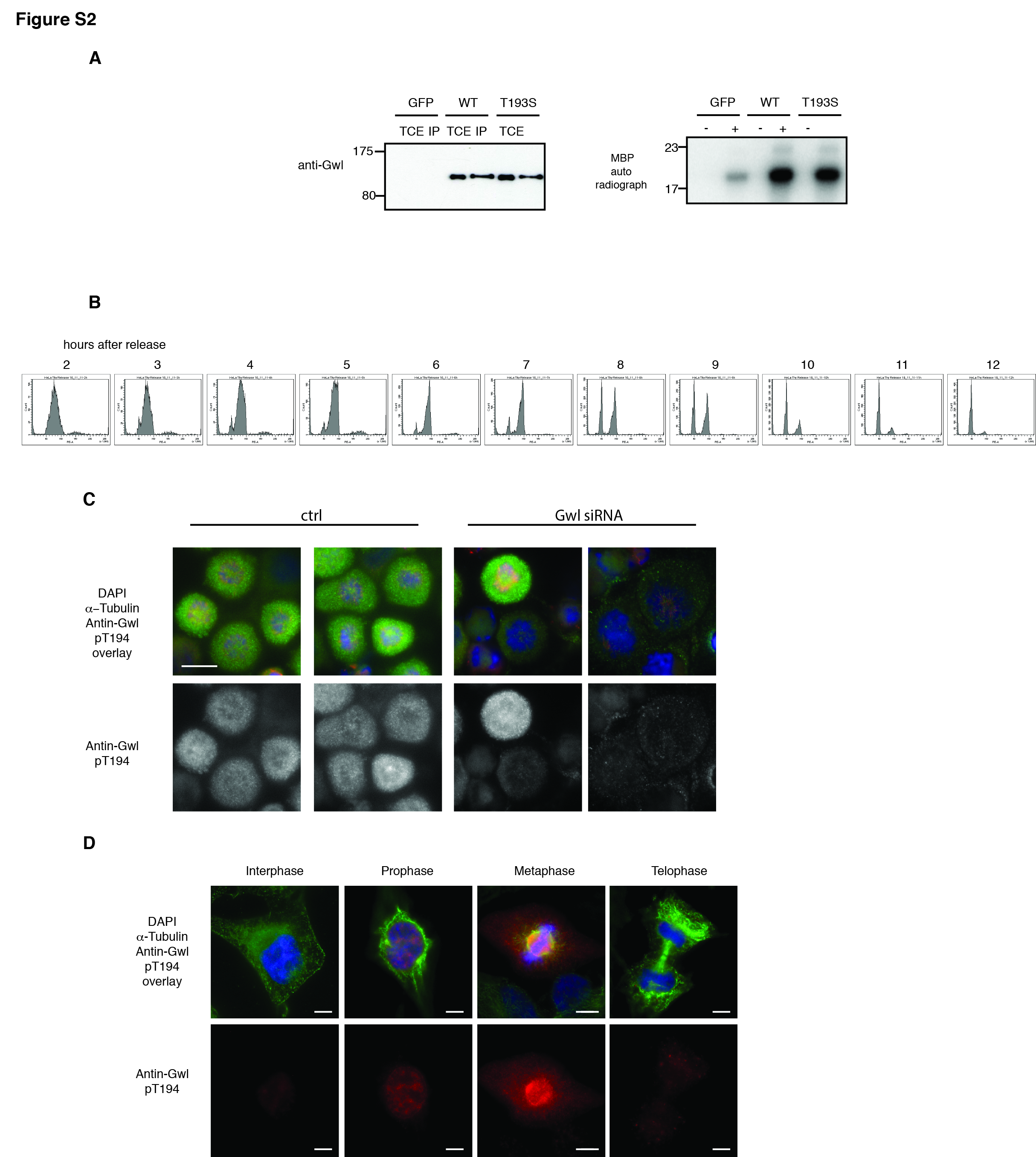

Supplement: Figure S2 — (A) WT and Thr194Ser Flag-Gwl were transiently expressed in 293T cells. 48 hours after transfection the cells were synchronized in mitosis by 18 hour incubation with nocodazole. Mutant and WT kinase were immuno-precipitated and assayed for kinase activity using MBP as a substrate. The kinase assays were analyzed by SDS PAGE and autoradiography. (B) FACS profile of thymidine release samples taken at indicated time points after thymidine washout. Cells passed through mitosis between 8 hours and 10 hours, correlating with Gwl Thr194 phosphorylation. (C) siRNA depletion of Gwl to determine specificity of the immuno-fluorescent signal of the phosphor Thr194 antibody. Cells were siRNA transfected with control and Gwl siRNA. 48 hours later, cells were treated with STLC to achieve mitotic arrest and stained with anti Gwl pThr194 (green), anti-αtubulin (red) and DAPI (blue). Note the mitotic cells that lost Gwl Thr194 signal in the knock-down sample. (D) Analysis of Gwl Thr194 phosphorylation by immunofluorescence with anti-Gwl pThr194 antibodies. DAPI staining and centrosome separation was used to identify mitotic cells at various stages. (TIF) [file pgen.1004004.s002.tif]

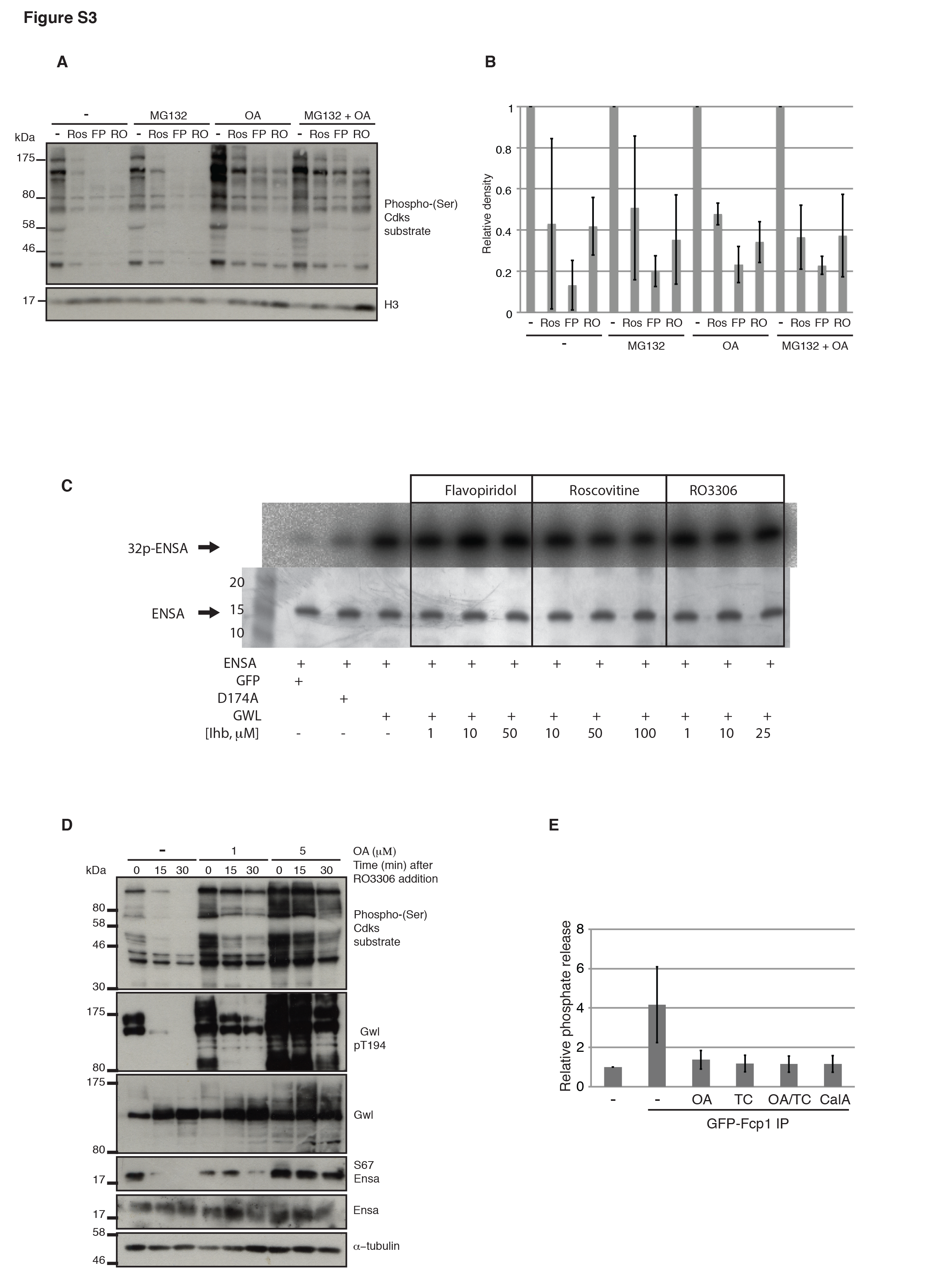

Supplement: Figure S3 — Mitotic exit triggered by Cdk inhibition. (A) Cells were arrested in mitosis by STLC and pretreated with 1 µM OA and 20 µM MG132 before inactivation of Cdk1 by 50 µM Roscovitine (Ros), 5 µM Flavopiridaol (Fp) and 10 µM RO3306 (RO). Mitotic exit was scored by measuring levels of phosphorylated SP by immune-blots. (B) Three independent experiments as shown in (A) were quantified using Image J. Error bars indicate standard deviation in the 3 data sets. (C) Effect of Cdk inhibitors on Gwl activity measured by IP/kinase assays. Flag-Gwl was transfected in 293T cells. 48 hours after transfection the cells were arrested in nocodazole for 18 hours. Flag-Gwl was immuno-precipitated from mitotic cells and incubated with recombinant Ensa/ARPP19 and γ32P ATP. The kinase assays were analyzed by SDS PAGE and autoradiography. (D) Effects of 1 and 5 µM OA on mitotic exit dephosphorylation. HeLa cells were synchronized in mitosis by Eg5 inhibition using 5 µM STLC and pretreated for one hour with 1 µM and 5 µM OA. Samples were taken for extraction and immunoblot analysis at indicated timepoints following treatment with 10 µM RO3306. (E) Effects of OA and TC on FCP1 phosphatase activity. Ensa phosphatase were performed as described in Figure 5 and the reactions were incubated with 1 µM OA, 10 µM TC and 100 nM CalA. (TIF) [file pgen.1004004.s003.tif]
